# Access to medicines through health systems in low- and middle-income countries

**DOI:** 10.1093/heapol/czz119

**Published:** 2019-12-09

**Authors:** Sachiko Ozawa, Raja Shankar, Christine Leopold, Samuel Orubu

**Affiliations:** 1 Medicines in Health Systems Thematic Working Group, Health Systems Global, Geneva, Switzerland; 2 Division of Practice Advancement and Clinical Education, UNC Eshelman School of Pharmacy, University of North Carolina, Chapel Hill, NC, USA; 3 Department of Maternal and Child Health, UNC Gillings School of Global Public Health, University of North Carolina, Chapel Hill, NC, USA; 4 Consulting Services, IQVIA, London, UK; 5 Division of Health Policy and Insurance Research, Harvard Medical School and Harvard Pilgrim Health Care Institute, Boston, MA, USA; 6 Institute for Health System Innovation and Policy, Boston, MA, USA; 7 Department of Pharmaceutics and Pharmaceutical Technology, Faculty of Pharmacy, Niger Delta University, Wilberforce Island, Bayelsa State, Nigeria

**Keywords:** Access, medicines, drugs, pharmaceuticals, developing countries, public health, health systems

## Abstract

Nearly 2 billion people globally have no access to essential medicines. This means essential medicines are unavailable, unaffordable, inaccessible, unacceptable or of low quality for more than a quarter of the population worldwide. This supplement demonstrates the implications of poor medicine access and highlights recent innovations to improve access to essential medicines by presenting new research findings from low- and middle-income countries (LMICs). These studies answer key questions such as: Can performance-based financing improve availability of essential medicines? How affordable are cardiovascular treatments for children? Which countries’ legal frameworks promote universal access to medicines? How appropriately are people using medicines? Do poor-quality medicines impact equity? Answers to these questions are important as essential medicines are vital to the Sustainable Development Goals and are central to the goal of achieving Universal Health Coverage. Access to affordable, quality-assured essential medicines is crucial to reducing the financial burden of care, preventing greater pain and suffering, shortening the duration of illness, and averting needless disabilities and deaths worldwide. This supplement was organized by the Medicines in Health Systems Thematic Working Group of Health Systems Global, a membership organization dedicated to promoting health systems research and knowledge translation. The five studies in the supplement further our understanding by showcasing recent successes and challenges of improving access to quality-assured medicines through health systems in LMICs.

**Key Messages**

- Nearly 2 billion people globally have no access to essential medicines.
- No access means medicines are unavailable, unaffordable, inaccessible, unacceptable or of low quality.
- We showcase successes and challenges of improving access to medicines in LMICs.

Nearly 2 billion people globally have no access to essential medicines—resulting in greater pain and suffering, prolonged illness, needless disabilities and preventable deaths (World Health Organization, 2017). Essential medicines are a vital component of the Sustainable Development Goals (SDGs) to ‘ensure access to safe, effective, quality and affordable essential medicines and vaccines for all (United Nations, 2019)’. Essential medicines are also central to the goal of achieving Universal Health Coverage (UHC), which ensures that all people have access to health services, including essential medicines, without risking financial hardship (World Health Organization, 2019). Access to affordable, quality-assured essential medicines is crucial to reducing the financial burden of care and improving population health worldwide.

Access to medicines in health systems encompasses five dimensions of access: (1) availability, (2) affordability, (3) accessibility (geographical availability), (4) acceptability (rational selection and use) and (5) quality (Wirtz *et al.*, 2016). Simply put, access to medicines implies that people have the right medicines of the right quality, at the right price and at the right place. This supplement addresses each of these five dimensions of access to medicines through new research studies from low- and middle-income countries (LMICs). The supplement was organized by the Medicines in Health Systems Thematic Working Group of Health Systems Global, a membership organization dedicated to promoting health systems research and knowledge translation (Mills, 2017; Health Systems Global, 2019). Based upon a call for papers, we publish here a collection of selected articles that showcase recent successes and challenges of improving access to quality-assured medicines through health systems in LMICs.

First, we present a study on the use of innovative financial incentives to drive medicine availability. Sieleunou *et al.* (2019) assessed whether performance-based financing (PBF) led to an improvement in perceived availability of essential medicines in Cameroon. By increasing autonomy of health facilities, enforcing regulation and having greater accountability of the medicine supply system, the case study suggests that the PBF programme was able to improve perceived availability of essential medicines. However, the authors also provide insights on the challenges faced, including payment delays, limited autonomy of health facilities, concerns around medicine quality, fragmentation of the drug management system and limited medicine accessibility in remote health facilities.

The second study examined the availability and affordability of cardiovascular medicines for children in Nigeria. Orubu *et al.* (2019) found that oral liquid formulations of medicines for children were not available, prices of generic medicines were high, and paediatric medicines that require compounding were unaffordable compared to government worker salaries. The study found that some generic oral tablets of cardiovascular medicines were 2–16 times more expensive than the International Reference Prices. Without age-appropriate oral liquid formulations of medicines for children, people incurred added costs of medicine compounding, making essential cardiovascular medicines for children unaffordable.

The third study examined the role of UHC laws in making essential medicines accessible. Perehudoff *et al.* (2019) identified and compared legal texts from national UHC legislation of 16 mostly LMICs: Algeria, Chile, Colombia, Ghana, Indonesia, Jordan, Mexico, Morocco, Nigeria, Philippines, Rwanda, South Africa, Tanzania, Turkey, Tunisia and Uruguay. The authors found that national laws toward medicines frequently did not include principles of good governance. Some legal texts also missed details on technical implementation such as sufficient government financing, and seeking international assistance and cooperation. This study provides examples of legal texts and an assessment tool—a checklist for evaluating national law and policy, and a wish list to guide legal reform—which can be used by national law makers in LMICs to promote universal access to essential medicines through UHC.

Another study considered the acceptability of medicines based on their rational selection and proper use. Luiza *et al.* (2019) assessed the pervasiveness of inappropriate use of medicines in Brazil, including non-adherence, un-authorized prescribers, and in-appropriate storage of medicines. Nearly half of the survey respondents engaged in at least one conduct of inappropriate medicine use, with many obtaining medicine prescriptions from non-formal sources. The most common profile of individuals inappropriately using medicines in the study were women, without free access to medicines, taking five or more medicines, without regular doctor visits, with two or more emergency visits in the previous year.

Finally, we include a study that examined the impact of medicine quality on equity. In a recent meta-analysis across LMICs, 13.6% of essential medicines were found to be substandard or falsified, including 12.4% for antibiotics and 19.1% for anti-malarials (Ozawa *et al.*, 2018). Evans *et al.* (2019) further found that substandard and falsified anti-malarials also widened health inequities where the burden of poor-quality malaria medicines predominantly fell on poor and rural populations in Uganda. The authors found that improving antimalarial quality can have a greater impact on reducing inequities compared to other interventions, such as replacing suboptimal treatments with Artemisinin Combination Therapies or removing antimalarial stock-outs.

The five studies together demonstrate the significant breadth of public health challenges of improving access to medicines through health systems in LMICs. These research findings support the five core challenges impacting essential medicines as highlighted by the Lancet Commission on Essential Medicines Policies: (1) inadequate financing to pay for a basket of essential medicines through UHC, (2) affordability of essential medicines, (3) assuring the quality and safety of essential medicines, (4) inappropriate use of essential medicines, and (5) developing and supplying new types of essential medicines that target unmet disease burden or offer more effective outcomes (Wirtz *et al.*, 2017). The research presented in this supplement provide new context to these challenges with specific examples from LMICs. The articles make several important contributions to the existing literature by answering questions about the impact of financial incentives on medicine access, examining the current status of medicine affordability and inappropriate use, offering an assessment tool to guide UHC legislation and linking medicine quality with equity.

One of the areas this supplement does not cover is the role of pharmaceutical companies in accelerating medicine access in LMICs. The Lancet Commission reveals that the present system for developing medicines is in crisis, failing to produce needed medicines that address the needs of millions of people globally (Wirtz *et al.*, 2017). For this area, we guide readers to the Access Observatory, which report existing programmes by pharmaceutical companies to improve access to disease prevention and treatment services in LMICs (Access Observatory, 2019). The Access to Medicines Index is another resource that ranks the world’s largest pharmaceutical companies based on their efforts to address access to medicines (Access to Medicine Foundation, 2019). Some progress has been made, but many challenges remain to promote global research and development of medicines for diseases that primarily affect populations in LMICs.

While access to medicines is part of the right to health as noted by the SDGs, many individuals struggle to access medicines. Despite the importance of medicines in reducing the world’s morbidity and mortality, availability of affordable quality-assured medicines continues to be poor, especially in LMICs. Ensuring access to medicines requires a complex systems approach involving diverse stakeholders and disciplines, from law and pharmacy to economics, with a global accountability mechanism (Simao *et al.*, 2018). It is essential to push existing boundaries to generate evidence and implement successful interventions to improve access to medicines through health systems in LMICs.
